# Performance of QuantiFERON-TB Gold test compared to tuberculin skin test in detecting latent tuberculosis infection in HIV- positive individuals in Iran

**DOI:** 10.4103/1817-1737.58959

**Published:** 2010

**Authors:** Masoud Mardani, Payam Tabarsi, Zohre Mohammadtaheri, Ehsan Chitsaz, Banafsheh Farokhzad, Fatemeh Hadavand, Latif Gachkar, Kambiz Nemati, Mohammad R. Masjedi

**Affiliations:** *Iranian Infectious Disease Research Center, Shaheed Beheshti Medical University, Tehran, Iran*; 1 *Mycobacteriology Research Center, NRITLD2, Shaheed Beheshti Medical University, Tehran, Iran*

**Keywords:** Diagnosis, Iran, smoking, tobacco, tuberculosis

## Abstract

**BACKGROUND:**

There is limited data about the performance of QuantiFERON-TB Gold (QFT-G) test in detecting latent tuberculosis infection (LTBI) in our region. We intended to determine the performance of QFT-G compared to conventional tuberculin skin test (TST) in detecting LTBI in HIV-positive individuals in Iran.

**METHODS:**

This study was conducted in a HIV clinic in Tehran, Iran in April 2007. A total of 50 consecutive HIV-positive patients, not currently affected with active tuberculosis (TB), were recruited; 43 (86%) were male. The mean age was 38 ± 7.2 years (21–53). All had history of Bacillus Calmette Guerin (BCG) vaccination. A TST with purified protein derivative (PPD) and whole-blood interferon-gamma release assay (IGRA) in reaction to ESAT-6 and CFP-10 antigens was performed and measured by enzyme-linked immuno-sorbent assay (ELISA). The agreement between TST and QFT-G results were analyzed using Kappa test.

**RESULTS:**

A total of 36 (72%) patients had negative and 14 (28%) revealed positive TST. For QFT-G, 20 (40%) tested positive, 19 (38%) tested negative, and the results in 11 cases (22%) were indeterminate. A total of 14 (28%) patients had a CD4 count of <200. Of the 14, TST + group, 12 had QFT-G +, only one case TST+/QFT-G-, and QFT-G was indeterminate in one TST positive case. Of the 36 patients with negative TST tests, 8 (22%) had positive GFT-G and 10 (28%) yielded indeterminate results. There was no association between a positive TST and receiving highly active anti-retroviral therapy (HAART) or absolute CD4 counts. Similarly, the association between QFT-G results and receiving HAART or CD4 counts was not significant (*P* = 0.06). Although TST results were not significantly different in patients with CD4 < 200 *vs*. CD4 >200 (*P* = 0.295), association between QFT-G results and CD4 cutoff of 200 reached statistical significance (*P* = 0.027). Agreement Kappa coefficient between TST and QFT-G was 0.54 (Kappa = 0.54, 95% CI = 38.4-69.6,*P* < 0.001).

**CONCLUSION:**

Detecting LTBI in HIV-positive individuals showed moderate agreement between QFT-G and LTBI in our study. Interestingly, our findings revealed that nontuberculous mycobacteria and prior BCG vaccination have minimal influence on TST results in HIV patients in Iran.

Tuberculosis (TB) has experienced a rising trend during the last few decades mostly attributed to the effects of newly emerging diseases, namely HIV. Also, in HIV-infected patients compared to immunocompetent individuals, latent tuberculosis infection (LTBI) is far more prone to develop into active TB.[1] Therefore, detecting LTBI, in high-risk groups such as HIV-infected individuals is one of the priorities in TB control.[2,3]

Currently, there is no gold standard for LTBI, and hence, diagnosis is defined according to the response to tuberculin skin test (TST), a test which yields both false positive with nontuberculous mycobacteria or Bacille Calmette-Guérin (BCG) vaccination and false negative results (due to anergy or immunosuppression).

However, as the current conventional approach, the TST does not satisfy the diagnostic needs of HIV-infected population and has been shown to be somewhat problematic in this group.[4–6]

TST did not already prove to have a high order of accuracy. In addition, the likelihood of positive TST in LTBI decreases in HIV-positive patients, mainly as a result of immunosuppression and anergy, which is apparently more common in CD4 counts below 100 cells/μl.[7] In addition, the protein purified derivatives (PPD) antigens used in TST frequently cross-react with those of many nontuberculous mycobcateria and this leads to a high rate of false-positive TST result. Moreover, TST may yield false-positive results in cases who have been vaccinated with BCG and this may last even for more than 15 years passed after the time of vaccination.[8,9]

Recently, and in response to such limitations, much attention has been paid to novel methods for LTBI detection. Interferon-gamma release assays (IGRAs) are new diagnostic methods that are found to be more effective and confer more accurate results in detecting active TB in immuncompetent individuals.[10,11]

QuantiFERON-TB Gold (QFT-G) test is a new whole-blood IGRA, which is now used in many countries.

However, the performance of QFT-G is not well established in detecting LTBI in HIV-infected people and a small number of studies have been conducted on this subject to date. Yet, the little available data on the utility of QFT-G is from studies carried out in different settings and is still controversial.[5,12]

So, doing more investigations on performance of QFT-G in persons with impaired immune systems, including persons with HIV/AIDS is declared as an important research need.[5,13,14]

In this study, we intended to determine the performance of QFT-G in detecting LTBI in HIV-positive people compared to the conventional TST test in Iran.

This cross-sectional study was conducted in a HIV clinic in Tehran, Iran from April 01 to April 30, 2007.

A total of 50 consecutive HIV-positive patients were recruited for the study. Patients who were not currently affected by active TB were included in the study. All patients had the history of being vaccinated by BCG. Of the 50 patients who entered the study, 43 (86%) were male and 7 (14%) were female. The mean age of the patients was 38 ± 7.2 years (Range = 21–53). A total of 12 (24%) patients had been receiving highly active antiretroviral therapy (HAART).

History taking and physical examination were performed by an expert physician. For all patients, a venipuncture was undertaken and 3 mm of whole blood was taken. For TST, 0.1 mm of PPD was administered intradermally. TST results were read and recorded after 48 hours by the same staff. Positive TST was defined as indurations >5 mm.

A whole-blood IGRA in reaction to Early Secreted Antigenic Target 6 (ESAT-6) and Culture Filtrate Protein 10 (CFP-10) antigens was performed and IFN-γ was measured via ELISA. According to the manufacturer's instructions, first, every patient's specimens-each containing 1 ml of whole blood-were poured into three separate tubes. The specimens were incubated in 37°C for 24 hours. Then the tubes were centrifuged in 2,000-3,000 rpm/min for 15 mins. The supernatant was frozen in −20° C. One tube served as the negative control containing only heparin. The second tube served as the positive control, with added phytohemagglutinin as the mitogen and the third was the test tube holding ESAT-6 and CFP-10 antigens. Gamma-interferon released in each tube was measured via ELISA and the measurements were read with QFT software. Indeterminate results, defined as a negative result in the positive control tube (mitogen tube), were identified.

Patient CD4 counts were retrieved from their records of routine follow-up and were entered in the patient's data sheet.

The obtained data were analyzed in SPSS 11.5 software. Agreement between the QFT-G and TST tests was calculated using Kappa Test. Indeterminate results were excluded from agreement analysis. The association between CD4 count and receiving HAART was analyzed with QFT-G and TST results. The values of Kappa lower than 0.4 were defined as poor, 0.41-0.6 as good and >0.6 as strong agreements.

A total of 36 (72%) patients had negative and 14 (28%) revealed positive TST. For QFT-G, 20 (40%) tested positive, 19 (38%) tested negative, and the results in 11 cases (22%) were indeterminate due to inadequate reaction to mitogen in the control tube. With respect to CD4 count, 14 (28%) had a CD4 count <200, 23 patients (46%) had a CD4 count of 200-350, 10 (20%) had a count of 350-500, and the remaining 3 (6%) had CD4 counts above 500.

Of the positive TST group, 12 had concomitant positive QFT-G, just one case was TST positive but QFT-G negative, and QFT-G was indeterminate in one TST positive case. Of the 36 patients with negative TST tests, 18 (50%) had negative QFT-G, 8 (22%) had positive GFT-G and 10 (28%) yielded indeterminate results [Table 1].

**Table 1:** QFT-G results with regard to CD4 count ranges

QFT-G	CD4: <200	CD4: 200–350	CD4: 350–500	CD4: >500
Positive	2	12	3	3
Negative	6	8	5	0
Intermediate	6	3	2	0
Total	14	23	10	3

There was no association between a positive TST result and receiving HAART. In addition, TST results did not show a statistically significant association with absolute CD4 counts [Table 2].

**Table 2:** QFT-G and TST results according to CD4 cutoff point <200

CD4 count	TST N. pts (% in column)		QFT-G N. pts (% in column)		
		
	Positive	Negative	Positive	Negative	Indeterminate
<200	2 (14.3)	12 (33.3)	2 (10)	6 (31.6)	6 (54.5)
≥200	12 (85.7)	24 (66.6)	18 (90)	13 (68.4)	5 (45.4)
Total	14 (28)	36 (72)	20 (40)	19 (38)	11 (22)
N. pts (% in row)					
*P* = 0.295			*P* = 0.027*		

* Level of significance is *P* < 0.05

Of the 20 positive QFT-G cases, 18 (90%) had CD4 counts above 200 while of the 11 indeterminate cases, 6 (55%) had CD4 counts below 200 [Table 3]. However, the association between QFT-G results and CD4 counts was not significant (*P* = 0.06). We also analyzed the CD4 counts with the cutoff point of 200 against TST and QFT-G results. TST results were not significantly different in patients with CD4 <200 and CD4 >200 (*P* = 0.295). However, the association between QFT-G results and CD4 cutoff of 200 reached statistical significance (*P* = 0.027). Likewise, there was not a significant association between receiving HAART and QFT-G results. Agreement Kappa coefficient between TST and QFT-G was 0.54 (Kappa = 0.54, 95% CI = 38.4-69.6,*P* < 0.001) [Table 3].

**Table 3:** QFT-G results with the corresponding TST results*

QFT-G	TST	
	
	Positive	Negative
Positive	12 (85.7)	8 (22.2)
Negative	1 (7.15)	18 (50)
Indeterminate	1 (7.15)	10 (27.8)
Total	14	36

* 12 (85.7%) with TST + revealed positive QFT-G favoring infection with rather than nontuberculous mycobacteria or previous BCG vaccination. QFT-G revealed positive results in 8 (22.2%) of patients with negative TST figures in parentheses are in percentage

## Discussion

According to available data from the World Health Organization, Iran is a country with moderate incidence for TB.[15] Early detection and treatment of LTBI, especially in high risk populations such as people living with HIV, are therefore significant concerns and a high priority in TB control planning.[5,14] So far, the performance of QFT-G in detecting LTBI in HIV patients is not fully investigated and the results are also controversial to some extent.

This may be ascribed to different TB prevalence as well as variable exposure to nontuberculous mycobacteria in various settings around the world.

In this study we attempted to compare of QFT-G with TST in detecting LTBI in the national referral center for TB in Iran.

Of the tested individuals, overall 28% had positive TST and 40% had positive QFT-G. This is a relatively high rate compared to other studies with 5% and 6% in report of S. Jones *et al*[5] and 10.9% and 14.8% in M. E. Balcells' *et al*.,[12] respectively.

This is at least in part can be attributed to the higher exposure to mycobacterial infections in Iran.

With regard to National Extended Program for Immunization (EPI) and according to personal histories, all patients in our study had inoculated BCG vaccination in their childhood, one of the issues that can interfere with the TST and contribute to false positive results. As MTB specific antigens are utilized in IGRA, unlike TST, QFT-G false positive results are uncommon. Interestingly, the majority of our patients who had positive TST results (86%) rendered positive QFT-G results with the exception of one negative QFT-G and one indeterminate QFT-G. This indicates that, in spite of prior BCG vaccination, which could potentially lead to false positive TST results in a large number of patients in Iran, a positive TST result was most probably due to exposure to mycobacterium TB rather than BCG vaccination.

It is shown that the effect of BCG vaccination on giving positive TST is of particular importance in the first 15 years after vaccination.[16] In Iran, BCG vaccine is inoculated at birth and all of our patients were 21 years old or more. So, this may be the rationale why BCG vaccination did not yield so many false positive TST results.

Also, this finding reveals that positive TST results in HIV-positive patients are mainly due to exposure/infection with MTB rather than nontuberculous mycobacteria since QFT-G as a specific test for MTB became positive in most cases when TST was positive.

On the other hand, TST results were negative in 8 (40%) of those cases with positive QFT-G results. Due to lack of a gold standard for LTBI, the specificity of IGRAs has been extrapolated by active TB and is found to be 98.1% in the immunocompetent population.[17] Therefore, given the high specificity of QFT-G, our findings denote a more favorable performance of of QFT-G in detecting exposure to MTB, compared with TST [Table 3].

Some studies have reported and compared the QFT-results with regard to CD4 count, as an index of patient's immune system status.[13,18] 90% of patients with a positive QFT-G result (N=18) had CD4 counts above 200. In contrast, 55% of cases with indeterminate QFT-G results (N=6) had CD4 counts below 200. This may be attributed to some degrees of immune system anergy in patients with low CD4 counts, which leads to irresponsiveness to mitogen and hence renders indeterminate QFT-G results.[13]

However, in our study, indeterminate QFT-G result occurred at 50% of the time in CD4 counts above 200, in contrast to S.Jones's *et al.* report that all indeterminate results occurred in CD4 below 200.

Another notable finding in our study was that there was no association between receiving HAART and TST results. Although HAART is proved to improve the immunity state of the patients and may potentially affect the immunologic tests, TST results were not significantly different between who received HAART and who did not.

This study demonstrated a moderate agreement between QFT-G and TST [76.9% (Kappa = 0.54, 95% CI = 38.4–69.6, *P* < 0.001)]. In addition, the study showed that almost all TST positive cases were detected by QFT-G; but, in turn, some QFT-G positive people had negative TSTs. Therefore, QFT-G has shown capable of determining nearly all TST positive cases and besides can find some other TST negative cases that properly react to QFT-G.

Overall, our study shows that in Iran, BCG vaccination and also other nontuberculous mycobacterial infections do not exert significant impact on TST results in Iranian HIV-infected patients.

We recommend that in middle- or low-income regions, where access to costly testing facilities may be limited and QFT-G cannot be used for screening of all HIV patients, TST be performed for all HIV-positive patients and if TST is negative then QFT-G be used to detect LTBI in HIV patients with high suspicion.

Our study also had some limitations: The sample size is relatively small in our study due to limited accessibility to QFT-G testing tools in our setting, which in turn, hampers interpreting the results as general conclusions. As well, the patients belong to an HIV clinic in Tehran, and thus, may not be the exact representative of the general population of HIV patients. Therefore, we suggest the findings of this preliminary study be investigated more comprehensively performing further studies especially in patients with CD4 counts below 200, with larger samples and with patients' recruitment in a multi-centre scale.

## Conclusion

QFT-G has been shown to be a more accurate tool to detect LTBI. Detecting LTBI in HIV-positive individuals showed moderate agreement between QFT-G and LTBI in our study. Interestingly, our findings revealed that nontuberculous mycobacteria and prior BCG vaccination have minimal influence on TST results in HIV patients in Iran. Further larger studies are required to elucidate the performance of QFT-G in LTBI detection in HIV patients in our local particularities in Iran.
